# Assessment of the Relationship between Carotid Intima-Media Thickening and Early-Stage Diabetic Kidney Disease Coupled with *Helicobacter pylori* Infection

**DOI:** 10.1155/2018/3793768

**Published:** 2018-04-16

**Authors:** Lei Feng, Changqing Deng, Yanxia Li

**Affiliations:** ^1^Graduate School, The Second Affiliated Hospital of Chongqing Medical University, Chongqing 400010, China; ^2^Emergency Department, Chongqing Steel Group General Hospital, Chongqing 400037, China; ^3^Hospital Infection Control Department, Suining Central Hospital, Suining 629000, China

## Abstract

**Objective:**

This study aimed to explore the associations between carotid intima-media thickness (CIMT) and early-stage diabetic kidney disease (DKD) coupled with *Helicobacter pylori (H. pylori)* infection in type 2 diabetic patients.

**Methods:**

A cross-sectional study including 180 type 2 diabetic participants was conducted to explore the associations between CIMT and early-stage DKD coupled with *H. pylori* infection, and a stepwise multivariate regression analysis evaluated the correlations of CIMT with clinical and serologic parameters.

**Results:**

The type 2 diabetic patients with early-stage DKD coupled with *H. pylori* infections had the highest CIMT values. Apolipoprotein B (ApoB), urine albumin/creatinine ratio (UACR), and interleukin-6 (IL-6) were independent predictors of CIMT.

**Conclusions:**

Early-stage DKD coupled with *H. pylori* infection may synergistically lead to significant CIMT thickening in type 2 diabetic patients. Additionally, ApoB, UACR, and IL-6 levels were important independent risk factors for increased CIMT.

## 1. Introduction

Atherosclerosis, a chronic inflammatory injury of the arterial wall, may lead to cardiovascular and cerebrovascular ischemic events, such as myocardial infarction and cerebral infarction [1–3]. Because atherosclerosis is highly prevalent worldwide, increasing numbers of research have been performed on the risk factors of atherosclerosis. Many studies have reported that increased CIMT is one of the risk factors for atherosclerosis, which alone can promote atherosclerosis progression [4–6]. Therefore, CIMT is considered a useful surrogate marker for atherosclerosis.

DKD, one of the most common complications in type 1 or type 2 diabetic patients and the major cause of end-stage renal disease worldwide, is characterized by proteinuria, decreased glomerular filtration rate, and deposition of extracellular matrix proteins [7, 8]. Previous studies have demonstrated that DKD is not only a progressive kidney disease but that it also affects multiple organ systems [9, 10]. For example, it might confer an increased risk of atherosclerosis, increasing cardiovascular morbidity and mortality [11]. However, results contradictory to the observations have also been reported. There have been several epidemiological reports suggesting no associations between DKD and CIMT [12, 13]. This is particularly true in the relationship between early-stage DKD and CIMT [14]. Although there is increased risk of ischemic stroke in DKD patients, DKD itself is not independently correlated with ischemic stroke [15].

*H. pylori* are spiral-shaped gram-negative bacteria and involved in the pathogenesis of several human diseases, including peptic ulcer, chronic gastritis, and gastric cancer [16]. Additionally, evidence has been provided that *H. pylori* infection is associated with atherosclerosis and cardiovascular and cerebrovascular disease, and it has been considered as one of the risk factors for atherosclerosis [17, 18]. Contradictory research findings have also suggested no association between *H. pylori* infection and CIMT or cardiocerebrovascular events [19, 20].

Therefore, the effects of early-stage DKD and *H. pylori* infection on CIMT, which is a marker of arteriosclerosis, are still debated. Many patients suffer from early-stage DKD accompanied by *H. pylori* infection. Whether their coexistence increases or decreases CIMT is unknown. Few studies have been performed on the relationship between CIMT and early-stage DKD coupled with *H. pylori* infection. Hence, we conducted the present study to investigate whether the effects of early-stage DKD coupled with *H. pylori* infection on CIMT are additive and to explore the correlations and differences in potentially influencing factors between atherosclerosis and early-stage DKD coupled with *H. pylori* infection. Furthermore, we provide practical recommendations for the early prevention and treatment of arteriosclerosis.

## 2. Materials and Methods

### 2.1. Participants

A total of 180 type 2 diabetic participants treated as outpatients or inpatients at Chongqing Steel Group General Hospital were recruited after signing the informed consent forms. All participants fulfilled the American Diabetes Association 2014 clinical diagnostic criteria for type 2 diabetes and did not accept dialysis. After physical, laboratory, and radiological examinations, no participants had signs of pregnancy, nondiabetic kidney disease, malignancy, heart failure, active infections, liver failure, or hepatocirrhosis. None of the participants in this study had experienced a previous cardiovascular event or received any organ transplantation. Among these participants, 89 participants were diagnosed with early-stage DKD characterized as UACR ≥ 30 to <300 mg/g and estimated glomerular filtration rate (eGFR) ≥ 60 mL/min/1.73 m^2^ [21, 22], 93 type 2 diabetic participants met the diagnostic criteria for *H. pylori* infection [23], and 49 type 2 diabetic participants met the diagnostic criteria for having neither DKD nor *H. pylori* infections. These 180 cases were categorized into 4 groups: 51 cases of early-stage DKD coupled with *H. pylori* infections (group A), 38 cases of early-stage DKD without *H. pylori* infections (group B), 42 cases of *H. pylori* infections without DKD (group C), and 49 cases of participants without DKD or *H. pylori* infections as controls (group D). A cross-sectional study was conducted to explore the associations between CIMT and early-stage DKD coupled with *H. pylori* infection in type 2 diabetic patients. Permission to perform the research was sought from the ethics committees of Chongqing Steel Group General Hospital, and the study protocol followed the tenets of the Declaration of Helsinki.

### 2.2. Ultrasonic Investigation of CIMT

CIMT analyses were carried out by two ultrasonologists, using a Doppler ultrasound machine (Siemens G50, Germany) with a 7.5 MHz transducer. Ultrasonologists carried out the examinations according to a standard protocol. The average CIMT was obtained from three independent measurements of the vertical distance from the edge of the first to the second echogenic line at 1.5 cm proximal to the carotid bifurcation in the bilateral common carotid arteries.

### 2.3. Detection of *H. pylori* Infection

A ^13^C-UBT test was carried out after 8 h of fasting. The ^13^C-urea breath test kits were obtained from Shenzhen Zhonghe Headway Bio-Sci & Tech Co. Ltd. The exhaled breath samples were obtained at baseline and 30 minutes after the oral intake of a ^13^C-urea capsule (75 mg). The infection status of the participant was determined by the use of ^13^C infrared spectrometry (Type YH08, Anhui Yanghe Medical Instrument Equipment Co. Ltd). A receiver operating characteristic analysis was used to calculate a cutoff value of delta over baseline (DOB). A DOB value ≥ 4 was considered positive, and a DOB value < 4 was considered negative.

### 2.4. Clinical and Biochemical Data

Detailed information regarding sex, age, body mass index (BMI), duration of diabetes, blood pressure, lifestyle-related risk factors, and medical history were obtained by our clinical specialists. Current smoker was defined as someone who had smoked at least 100 cigarettes throughout one's life and currently smokes cigarettes [24]. Urine and fasting venous blood samples were collected from participants in the morning and sent immediately to clinical laboratories for measurement. Serum levels of glucose, glycosylated haemoglobin (HbA1C), C-reactive protein (CRP), renal function indicators, blood lipids, and apolipoproteins were determined using an automatic biochemical analyzer (Beckman Coulter AU5800, Japan). eGFR was calculated with the use of the modification of diet in the renal disease formula: eGFR (mL/min/1.73 m^2^) = 186 × (serum creatinine × 0.011)^−1.154^ × (age)^−0.203^ × (0.742 for women) [22]. Urinary albumin and creatinine were determined by using the rate nephelometry and sarcosine oxidase method. UACR was calculated as follows: UACR (mg/g) = urinary albumin/urinary creatinine. Serum levels of malondialdehyde (MDA) and IL-6 were determined using an enzyme-linked immunosorbent assay. The enzyme-linked immunosorbent assay kits for MDA and IL-6 were obtained from Shanghai Enzyme-linked Biotechnology Co., Ltd., Shanghai, China. All tests were carried out according to the manufacturer's protocol.

### 2.5. Statistical Analysis

The clinical characteristics and biochemical data of each of the four groups were presented as the sample size and percentages (%) for categorical variables and expressed as mean ± standard error of the mean (SEM) for quantitative variables. Categorical variables were compared by the use of the chi-square (*χ*^2^) test. One-way ANOVAs were used in the analysis of multiple sample means. For post hoc multiple comparisons between groups, variables were analyzed with the use of the LSD test or Tamhane's test. Multivariable analyses of the association between CIMT and the other parameters were performed by using a stepwise multivariate regression analysis. All data analyses were conducted using SPSS for Windows version 22.0. *P* values < 0.05 were considered statistically significant.

## 3. Results

### 3.1. Characteristics of Participants

Demographic and clinical data of the study participants are summarized in Table 1. Groups were compared for any statistical differences in age, sex, BMI, smoking habit, CIMT, kidney factors, diabetes factors, hypertension prevalence, blood lipids, blood apolipoproteins, and inflammatory cytokines. There were no differences in age, sex, BMI, smoking habit, hypertension prevalence, duration of diabetes, fasting plasma glucose (FPG), and HbA1C among the four groups. However, participants in group A had increased CIMT as compared to the participants of the other groups. Compared with the participants in groups C and D, participants in groups A and B had significantly higher UACR and lower eGFR. Additionally, significant differences were detected for serum triglycerides (TGs), total cholesterol (TC), high-density lipoprotein (HDL), and low-density lipoprotein (LDL) levels among the groups. The levels of serum TC were significantly higher in groups C and D than in groups A and B, and the levels of serum TGs were significantly higher in groups A and B than in groups C and D. The levels of serum LDL were significantly higher in group C than in groups B and D, and the levels of serum HDL were significantly higher in groups A and C than in group D. No differences were observed in apolipoprotein A1 (ApoA1) levels. However, the serum ApoB levels in group A were significantly higher than that in the other groups. Serum CRP, IL-6, and MDA levels were determined in each of the groups. The levels of serum CRP were significantly higher in groups A and C than in groups B and D, and the levels of serum IL-6 were significantly higher in group A than in groups B and D. However, no differences in serum MDA levels were observed among the four groups.

### 3.2. Correlations of CIMT and the Other Parameters in Participants

We used CIMT as a dependent variable in a stepwise multivariate regression analysis, to examine its associations with all related independent variables. We found that ApoB, UACR, and IL-6 were independent and powerful predictors of CIMT (Table 2).

## 4. Discussion

To our knowledge, the relationship between CIMT and early-stage DKD coupled with *H. pylori* infection has not been described before. Our study shows that the coexistence of early-stage DKD and *H. pylori* infection leads to a significant CIMT increase, exerting a truly synergistic effect on atherosclerosis development in type 2 diabetic patients. DKD, a microvascular complication of longstanding diabetes mellitus, is characterized by initially albuminuria and eventually renal failure [25]. Several previous studies have demonstrated that DKD is also associated with CIMT [26]. In addition, the severity of DKD has been reported to be positively correlated with CIMT, and DKD should therefore be considered as another probable independent risk factor that contributes to atherosclerosis development [27]. However, contradictory results in the current literature suggest that patients with DKD do not have increased CIMT, particularly so in patients with early-stage DKD [12]. Consistent with these results, no association was found between early-stage DKD and CIMT in our study. A prospective cohort study demonstrated that *H. pylori* infection can increase CIMT and promote carotid plaque instability [28]. It has also been reported recently that *H. pylori*-positive subjects tend to have higher CIMT than *H. pylori*-negative subjects and *H. pylori* infection might play an important role in stroke, atherosclerosis, and other cardiovascular diseases [29, 30]. However, previous publications had reached opposing conclusions, indicating that *H. pylori* infection was not a major risk factor for atherosclerosis on the basis of CIMT measurements [31, 32]. The data in our study support the opposite viewpoint. In addition, we provided the first evidence that type 2 diabetic patients with early-stage DKD coupled with *H. pylori* infections appeared to have higher CIMT than type 2 diabetic patients with early-stage DKD or *H. pylori* infections. Furthermore, a stepwise multivariate regression analysis confirmed the independent risk factors for CIMT, including ApoB, UACR, and IL-6. In summary, type 2 diabetic patients with early-stage DKD coupled with *H. pylori* infections might have greater CIMT, resulting in an increased incidence of atherosclerosis.

The exact mechanisms as to how the combination of early-stage DKD and *H. pylori* infection might lead to an increase in CIMT are unknown. Both proteinuria and decreased eGFR are important risk factors for atherosclerosis and are defined as the clinicopathologic characteristics of early-stage DKD. Our data indicated that in type 2 diabetic patients with early-stage DKD or early-stage DKD coupled with *H. pylori* infections, a significant increase in UACR and a significant decrease in eGFR were observed. However, there were no differences in UACR and eGFR between type 2 diabetic patients with *H. pylori* infection alone and controls. Additionally, the regression analysis indicated that UACR was positively correlated with CIMT. These findings support the notion that proteinuria of early-stage DKD is a risk factor for atherosclerosis, which might play an important role in increasing CIMT in type 2 diabetic patients.

Apolipoproteins and blood lipids are also significant risk factors for atherosclerosis. Previous studies showed that TG and HDL cholesterol levels are associated with a greater CIMT and the risk of future adverse cardiovascular events [33]. A large number of studies have demonstrated that DKD and *H. pylori* infection may contribute to dyslipidemia by affecting ApoA1, ApoB, TG, TC, HDL, LDL, and oxidized LDL levels, and that these effects may promote the development of atherosclerosis [34–36]. In this study, we found that apolipoproteins and blood lipid levels, including serum TG, HDL, and ApoB levels, were highest in type 2 diabetic patients with early-stage DKD coupled with *H. pylori* infections. The regression analysis identified that ApoB was also positively related to CIMT. These findings indicated that lipid metabolic disorders resulted from the coexistence of early-stage DKD and *H. pylori* infection, which could contribute to an increase in CIMT.

Atherosclerosis is defined as a chronic inflammatory disease. A large number of inflammatory cytokines have been implicated in the development of atherosclerosis [37, 38]. Many studies have reported that patients with DKD present higher serum levels of IL-6 and CRP [39]. In addition, other studies have demonstrated that *H. pylori* infection is associated with persistent low-grade systematic inflammation, which can stimulate the production of inflammatory cytokines including IL-6, IL-18, and CRP [40, 41]. Our data here showed that participants with early-stage DKD coupled with *H. pylori* infections had the highest serum levels of IL-6, and the serum levels of CRP were significantly higher in participants with *H. pylori* infection. The regression analysis also found that IL-6 was positively associated with CIMT. These data indicated that the coexistence of early-stage DKD and *H. pylori* infection might act synergistically to produce inflammatory cytokines, promoting the development of atherosclerosis. Atherosclerosis also can be induced by oxidative stress [42]. MDA is produced by lipid peroxidation reactions and a marker of oxidative stress [43]. While many studies have demonstrated increased serum levels of MDA in patients with DKD or *H. pylori* infection [44, 45], no differences were observed in serum MDA levels in our study. Therefore, the correlations between oxidative stress and CIMT in type 2 diabetic patients with early-stage DKD coupled with *H. pylori* infections remain undefined and require further research.

There are several limitations to our study that should be noted. First, our sample population was relatively small and biased because participants in the study were only selected from those who visited a designated hospital. Second, several confounders, such as drug use and family history of cardiovascular diseases, were not considered. Therefore, larger samples and further evaluation of potential confounders will be required in prospective studies.

In summary, we show that the coexistence of early-stage DKD and *H. pylori* infection can lead to marked proteinuria, lipid metabolic disorders, and increased serum levels of inflammatory cytokines and then has synergistic effects on CIMT in type 2 diabetic patients. Hence, we suggest that eradicating *H. pylori* infections and avoiding the development of DKD are important measures for the prevention and treatment of cardiocerebrovascular disease in type 2 diabetic patients.

## Figures and Tables

**Table 1:** Demographic and clinical data of the study participants.

	Group A	Group B	Group C	Group D	Chi-square or *F*	*P*
*N*	51	38	42	49		
Age (years)	46.10 ± 0.58	46.79 ± 0.63	46.64 ± 0.54	46.61 ± 0.53	0.292	0.831
Gender (male/female)	41/10	30/8	33/9	38/11	0.124	0.989
Current smoker (%)	23.53 (12/51)	21.05 (8/38)	23.81 (10/42)	22.45 (11/49)	0.109	0.991
CIMT (mm)	0.84 ± 0.009^#△▲^	0.76 ± 0.013	0.75 ± 0.011	0.75 ± 0.009	18.007	<0.001
BMI (kg/m^2^)	25.57 ± 0.20	25.58 ± 0.24	25.90 ± 0.22	25.45 ± 0.19	0.818	0.486
eGFR (mL/min/1.73 m^2^)	99.31 ± 1.64^#◇^	100.12 ± 2.20^△▲^	107.47 ± 1.70	107.54 ± 1.35	7.146	<0.001
UACR (mg/g)	100.46 ± 1.92^#◇^	98.18 ± 2.42^△▲^	10.45 ± 0.29	10.37 ± 0.24	1165.8	<0.001
FPG (mmol/L)	7.18 ± 0.06	7.19 ± 0.07	7.34 ± 0.06	7.34 ± 0.06	1.976	0.119
HbA1C (%)	7.46 ± 0.07	7.39 ± 0.07	7.26 ± 0.05	7.26 ± 0.09	1.932	0.126
Duration of diabetes (years)	10.22 ± 0.35	10.03 ± 0.29	9.31 ± 0.23	9.76 ± 0.26	1.745	0.16
Hypertension (%)	33.33 (17/51)	31.58 (12/38)	26.19 (11/42)	30.61 (15/49)	0.581	0.901
TGs (mmol/L)	1.85 ± 0.04^#◇^	1.85 ± 0.04^△▲^	1.72 ± 0.04	1.70 ± 0.04	4.011	0.009
TC (mmol/L)	4.57 ± 0.04^#◇^	4.55 ± 0.05^△▲^	4.76 ± 0.05	4.71 ± 0.05	4.612	0.004
LDL (mmol/L)	2.57 ± 0.02	2.51 ± 0.02	2.63 ± 0.03^#◇^	2.51 ± 0.02	5.152	0.002
HDL (mmol/L)	1.47 ± 0.03	1.41 ± 0.03	1.47 ± 0.03	1.38 ± 0.03^△▲^	2.743	0.045
ApOA1 (g/L)	1.50 ± 0.02	1.47 ± 0.03	1.51 ± 0.03	1.46 ± 0.03	0.885	0.45
APOB (g/L)	1.17 ± 0.02^#△▲^	1.01 ± 0.03	1.08 ± 0.03	1.07 ± 0.03	5.388	0.001
IL-6 (ng/L)	35.10 ± 0.59^#◇^	31.85 ± 0.64	33.45 ± 0.74	31.99 ± 0.56	6.126	0.001
CRP (ng/L)	5.44 ± 0.11^#◇^	4.41 ± 0.15	5.13 ± 0.11^△▲^	4.65 ± 0.11	14.65	<0.001
MDA (ng/L)	11.53 ± 0.67	12.13 ± 0.82	11.21 ± 0.75	12.58 ± 0.83	0.646	0.586

CIMT = carotid intima media thickness; BMI = body mass index; eGFR = estimated glomerular filtration rate; UACR = urine albumin/creatinine ratio; FPG = fasting plasma glucose; HbA1C = glycosylated haemoglobin; TGs = triglycerides; TC = total cholesterol; LDL = low-density lipoprotein; HDL = high-density lipoprotein; ApoA1 = apolipoprotein A1; ApoB = apolipoprotein B; IL-6 = interleukin-6; CRP = C-reactive protein; MDA = malondialdehyde. CIMT: ^#^*P* < 0.001 (group A versus group B); ^△^*P* < 0.001 (group A versus group C); ^▲^*P* < 0.001 (group A versus group D). eGFR: ^#^*P* = 0.001 (group A versus group C); ^◇^*P* < 0.001 (group A versus group D); ^△^*P* = 0.005 (group B versus group C); ^▲^*P* = 0.003 (group B versus group D). UACR: ^#^*P* < 0.001 (group A versus group C); ^◇^*P* < 0.001 (group A versus group D); ^△^*P* < 0.001 (group B versus group C); ^▲^*P* < 0.001 (group B versus group D). TGs: ^#^*P* = 0.021 (group A versus group C); ^◇^*P* = 0.008 (group A versus group D); ^△^*P* = 0.031 (group B versus group C); ^▲^*P* = 0.013 (group B versus group D). TC: ^#^*P* = 0.005 (group A versus group C); ^◇^*P* = 0.028 (group A versus group D); ^△^*P* = 0.004 (group B versus group C); ^▲^*P* = 0.019 (group B versus group D). LDL: ^#^*P* = 0.001 (group C versus group B); ^◇^*P* = 0.001 (group C versus group D). HDL: ^△^*P* = 0.015 (group D versus group A); ^▲^*P* = 0.029 (group D versus group C). APOB: ^#^*P* < 0.001 (group A versus group B); ^△^*P* = 0.032 (group A versus group C); ^▲^*P* = 0.011 (group A versus group D). IL-6: ^#^*P* < 0.001 (group A versus group B); ^◇^*P* < 0.001 (group A versus group D). CRP: ^#^*P* < 0.001 (group A versus group B); ^◇^*P* < 0.001 (group A versus group D); ^△^*P* < 0.001 (group C versus group B); ^▲^*P* = 0.005 (group C versus group D).

**Table 2:** Results of a stepwise regression analysis with the use of CIMT as the dependent variable and the related covariates as independent variables.

Independent variable	Nonstandardized coefficients		Standardized coefficients	*t*	*P*
	*B*	Standard error			
Constant	0.353	0.020	17.735	<0.001	
ApoB	0.309	0.016	0.738	18.900	<0.001
UACR	0.001	<0.001	0.308	9.377	<0.001
IL-6	0.002	0.001	0.097	2.481	0.014
